# Characterization of Wnt signaling pathway under treatment of *Lactobacillus acidophilus* postbiotic in colorectal cancer using an integrated in silico and in vitro analysis

**DOI:** 10.1038/s41598-023-50047-x

**Published:** 2023-12-27

**Authors:** Nafiseh Erfanian, Saeed Nasseri, Adib Miraki Feriz, Hossein Safarpour, Mohammad Hassan Namaei

**Affiliations:** 1 Student Research Committee, Birjand University of Medical Sciences, Birjand, Iran; 2 Cellular and Molecular Research Center, Birjand University of Medical Sciences, Birjand, Iran; 3 Infectious Diseases Research Center, Birjand University of Medical Sciences, Birjand, Iran

**Keywords:** Cancer, Cancer genomics

## Abstract

Colorectal cancer (CRC) is a prevalent and life-threatening cancer closely associated with the gut microbiota. Probiotics, as a vital microbiota group, interact with the host’s colonic epithelia and immune cells by releasing a diverse range of metabolites named postbiotics. The present study examined the effects of postbiotics on CRC’s prominent differentially expressed genes (DEGs) using in silico and in vitro analysis. Through single-cell RNA sequencing (scRNA-seq), we identified four DEGs in CRC, including secreted frizzled-related protein 1 (*SFRP1*), secreted frizzled-related protein 2 (*SFRP2*), secreted frizzled-related protein 4 (*SFRP4*), and matrix metallopeptidase 7 (*MMP7*). Enrichment analysis and ExpiMap, a novel deep learning-based method, determined that these DEGs are involved in the Wnt signaling pathway as a primary cascade in CRC. Also, spatial transcriptome analysis showed specific expression patterns of the *SFRP2* gene in fibroblast cell type. The expression of selected DEGs was confirmed on CRC and normal adjacent tissues using Real-Time quantitative PCR (RT-qPCR). Moreover, we examined the effects of postbiotics extracted from *Lactobacillus acidophilus* (*L. acidophilus*) on the proliferation, migration, and cell cycle distribution of HT-29 cells using MTT, scratch, and flow cytometry assays. Our results showed that *L. acidophilus* postbiotics induce cell cycle arrest at G1 phase and also had anti-proliferative and anti-migration effects on HT-29 cells, while it did not exert anti-proliferative activity on control fibroblasts. Finally, we revealed that treating HT-29 cells with postbiotics can affect the expression of selected DEGs. We suggested that *L. acidophilus* postbiotics have therapeutic potential in CRC by modulating key genes in the Wnt pathway.

## Introduction

Colorectal cancer (CRC) is a highly prevalent malignancy that stands as the third most prevalent cancer globally in terms of incidence^1^. The role of environmental factors, genetic mutations, and epigenetic changes in the onset and advancement of CRC is widely recognized. However, the emergence of advanced multi-omics technologies has revealed compelling evidence highlighting the significant association between gut microbiota and the growth and advancement of CRC^2^. The gut microbiome is a complex community of microorganisms that have an essential role in maintaining the gut’s and body’s overall health. Dysbiosis of the gut microbiota, in particular, has significantly contributed to many cancers, mainly gastrointestinal (GI) ones. Many studies have shown that individuals with CRC have a distinct gut microbiome compared to healthy individuals. Specifically, these studies suggest a reduction in the abundance and diversity of beneficial bacteria and an increase in the prevalence of harmful bacteria within the gastrointestinal tract^3,4^. This imbalance in the gut microbiota has been linked to inflammation and other processes that contribute to the development of CRC^5,6^.

In this way, some studies highlight the potential involvement of specific bacterial strains in CRC progression via intricate signaling pathways. For instance, *Fusobacterium nucleatum* has been shown to promote tumor growth by activating the Wnt signaling pathway while concurrently dampening adaptive immunity mediated by CD3+ T cells^7^. Similarly, *Bacteroides fragilis* intricately engages multiple signaling pathways within colonic epithelial cells, including NF-κB, Wnt, and MAPK, which in turn stimulate the production of inflammatory mediators that significantly contribute to the progression of CRC. Conversely, certain bacterial species, such as those known for their butyrate-producing abilities, demonstrate a remarkable ability to inhibit inflammation and carcinogenesis^8^. Studies have demonstrated the ability of butyrate to attenuate the proliferation of human colon cancer cells and promote apoptosis by downregulating c-Myc and regulating p57 levels^9^. It also induces apoptosis in CRC^10^. Multiple lines of evidence support butyrate’s inhibitory actions on inflammation and carcinogenesis. These actions encompass inhibiting the production of proinflammatory mediators^8^, modulating NF-kB activation and histone deacetylation^11^, and downregulating the Wnt signaling pathway^12^.

In light of the potential involvement of specific bacterial strains in CRC development and the intriguing role of certain bacteria, there is growing interest in strategies to correct gut microbiota imbalances. One potential strategy for correcting the imbalance in the gut microbiota is through the use of probiotics^13,14^. Probiotics are living microorganisms with potential anti-cancer properties, indicating their potential to constrain or prevent the growth of cancer cells^15^. Another recent method for rectifying the gut microbiota imbalance is fecal microbiota transplantation, which has enhanced the therapeutic benefits for patients with various diseases, including CRC. However, how the disturbed or recovered intestinal microbiota influences the development of diseases remains unclear^16^.

While existing studies have shed light on how using probiotic bacteria influences processes like carcinogenesis, using live bacteria in cancer patients raises concerns about potential risks of infection or immune suppression^13^. Recently, postbiotics, bioactive compounds generated by probiotic bacteria during their life cycle or after their lysis or death, presented a promising alternative to probiotics^17,18^. Postbiotics could enhance the production of mucins, antimicrobial proteins (bacteriocins and peptides), cytokines (Interleukin 10 and 18), and neurotransmitters (serotonin) in the intestine to main the gut microbiota, intestinal barrier system and other immune functions due to having valuable ingredients such as short-chain fatty acids (SCFAs) (acetate, propionate, butyrate, etc.). Although these characteristics make them promising tools for the inhibition and adjuvant treatment of many diseases, their mechanism of action is not fully understood^19,20^.

Recent progressions in single-cell RNA sequencing (scRNA-seq) technology enable us to understand better cancer evolution, tumor heterogeneity, and the tumor microenvironment (TME)^21–23^. In this study, we tried to weigh the various signaling pathways associated with CRC using scRNA-seq. Then, we evaluated the relative expression of candidate genes via *Lactobacillus acidophilus* (*L. acidophilus*) postbiotics effects on the HT-29 cell line.

## Materials and methods

**Figure 1:**
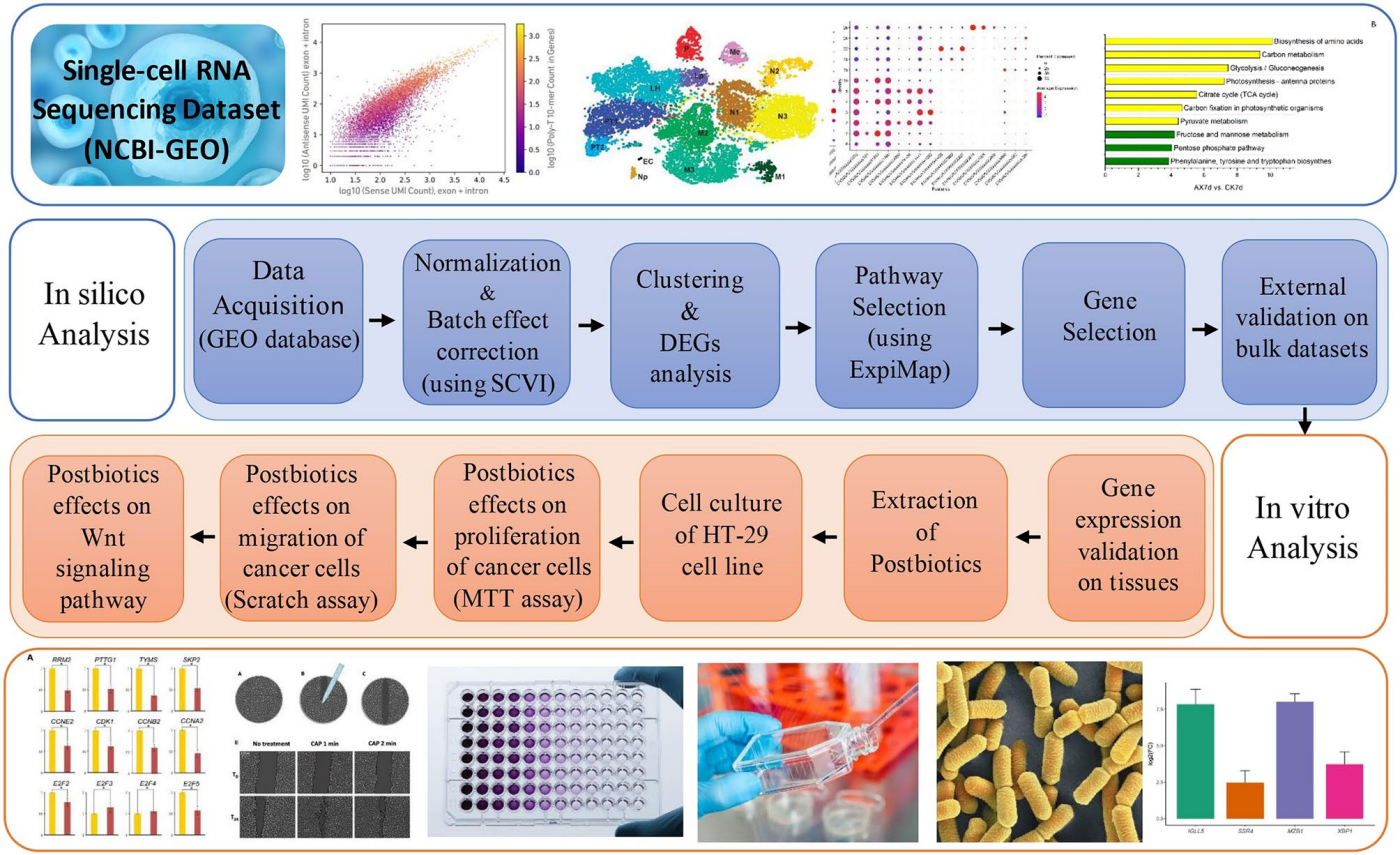
The overall procedure’s workflow.

The workflow of the entire procedure is presented in (Fig. 1). Under the ethical guidelines; this research study was conducted following the approved ethics ID IR.BUMS.REC.1400.332 was issued by the Birjand University of Medical Science Ethics Committee, Iran.

### In-silico analysis

#### scRNA-seq data analysis of online dataset

The scRNA-seq raw data for this study was gained from the experiment by Zheng et al., with accession number “GSE161277” (https://www.ncbi.nlm.nih.gov/geo/query/acc.cgi?acc=GSE161277). scRNA-seq was conducted on 11 tissue samples collected from four adenoma, four carcinoma, and three normal tissues. (Gene symbols were added to the GEO expression matrix using data from the GPL20795 HiSeq X Ten). We eliminated low-quality cells by applying specific criteria: a minimum number of reads greater than 750, a minimum number of genes greater than 200, and a maximum fraction of mitochondrial reads below 0.2. The data was then normalized and transformed logarithmically using the scanpy package (v1.9.1) through the functions scanpy.pp.normalize_per_cell (counts_per_cell_after = 10e4) and scanpy.pp.log1p.

To address any batch effects, we employed a probabilistic approach that considers different sources of variation in an unsupervised manner. This was achieved by employing a deep generative modeling technique called scVI^24^. Essentially, each cell’s transcriptome underwent a nonlinear transformation to obtain a low-dimensional, batch-corrected, latent embedding. We executed the scVI algorithm (0.5.0) with default parameters (n_hidden = 128, n_latent = 30, n_layers = 2, dispersion = ‘gene’) on all cells that passed the quality control, treating each sample as an individual batch.

Next, we employed the scanpy.pp.highly_variable_genes function to identify highly variable genes. Principal Component Analysis (PCA), neighborhood graph construction (scanpy.pp.neighbors), and Uniform Manifold Approximation and Projection (UMAP) were subsequently computed. For automatic annotation, we employed the celltypist package (v1.2.0) and utilized the celltypist.annotate function (model = ‘Immune_All_High.pkl’, majority_voting = True, min_prob = 3)^25,26^.

#### Functional pathway analysis of DEGs and target gene selection for experimental validation

To distinguish between healthy and tumoral tissues, we utilized the sc.tl.rank_genes_groups function and employed the t-test method to analyze differentially expressed genes (DEGs). DEGs were chosen for subsequent analysis if they met the criteria of an adjusted p-value < 0.05 and a log fold change > |2|. The p-values were adjusted using the Benjamini–Hochberg method.

We utilized the web-based tool Enrichr (available at https://maayanlab.cloud/Enrichr/) to understand DEGs’ biological roles and signaling pathways. This tool allowed us to conduct gene ontology function analysis, explicitly focusing on biological processes and pathway enrichment analyses.

Additionally, we employed expiMap^27^, a deep-learning model inspired by biology, to facilitate single-cell reference mapping. Using expiMap with default parameters, we could map individual cells onto comprehensible biological components corresponding to established “gene programs”. This approach enabled us to simultaneously learn and refine the activity of each cell within these gene programs and discover entirely new programs.

Lastly, we specifically focused on the signaling pathway that exhibited shared characteristics identified through enrichment analyses and expiMap output lists for the subsequent analysis. Through the enrichment analysis, we carefully selected pivotal DEGs for experimental validation to evaluate their expression patterns when subjected to postbiotics treatment.

#### Cell–cell interaction analysis

CellPhoneDB toolkit was used, which is capable of handling a substantial volume of interacting pairs (over 100,000) and cluster combinations (over 100). In this research, the approach was applied with 1000 permutations, focusing only on interactions where both ligand and receptor genes were expressed in at least 10% of the cells.

#### Characterization of selected genes across spatial transcriptome data

We examined a 500-gene panel of spatial transcriptome data from Vizgen MERSCOPE. In particular, MERSCOPE data is from human colon cancer (https://console.cloud.google.com/storage/browser/vz-ffpe-showcase/HumanColonCancerPatient2;tab=objects?pageState=(%22StorageObjectListTable%22:(%22f%22:%22%255B%255D%22))&prefix=&forceOnObjectsSortingFiltering=false&pli=1) accessed on 12 June 2023. By clustering cells based on gene expression and remapping these groups onto a spatial representation of the tumor, we were able to create a molecular and cellular atlas of each of these unique patient tumors using the Vizgen Cell Boundary Stain kit.

#### Characterization of selected DEGs across external datasets

For studying the expression patterns of our selected genes, we conducted DEG analysis on the GSE156451 dataset, which consists of bulk RNA-seq data and is publicly available through the Gene Expression Omnibus (GEO) database (https://www.ncbi.nlm.nih.gov/geo/geo2r/?acc=GSE156451). From the total of 144 samples within the dataset, we handpicked 47 tumor samples and 48 adjacent specimens for further analysis using the GEO2R software.

Additionally, to confirm the reliability of selected genes, we employed the Gene Expression Profiling Interactive Analysis (GEPIA) platform (http://gepia.cancer-pku.cn). GEPIA is a web-based application specifically designed for the comparative analysis of gene expression in cancer and normal tissues. Using this tool, we generated box plots based on colon adenocarcinoma (COAD) gene expression data, utilizing Anova analysis on log2 (TPM + 1) values, with a |Log2FC| cut-off of 1. Furthermore, to assess the impact of the DEGs on the overall survival of CRC patients, survival analysis was made through the GEPIA online database.

### In vitro analysis

#### Absolute RT-qPCR of total *L. acidophilus* load

Healthy individuals and individuals diagnosed with CRC underwent colonoscopy procedures administered by gastroenterologists. CRC cases identified as positive during the initial screening were subsequently confirmed through histopathology tests. A total of 50 fecal samples, comprising 25 CRC patients and 25 healthy subjects, were meticulously selected. Inclusion and exclusion criteria were applied based on specific medical histories and medication usage. These fecal samples were carefully collected and then preserved at − 80 °C. Standard strains of *Lactobacillus acidophilus* were cultured. Genomic DNA was extracted from fecal specimens using the FavorPrep Stool DNA Isolation Mini Kit, produced by Favorgen in Taiwan, and the standard strain method, which involved phenol–chloroform extraction. The quantification of bacterial expression copy numbers per gram of feces was determined using Absolute RT-qPCR, relying on standard curves, with specific primers detailed in Table 1.

**Table 1:** Primer sequences.

Primer	Sequence
*SFRP1*	F: CTTCTACTGGCCCGAGATGCT
	R: ATGGCCTCAGATTTCAACTCGT
*SFRP2*	F: AGCCCGACTTCTCCTACAAGC
	R: CTTCATGACCAGCGGGATCCA
*SFRP4*	F: ACAAATTCTTCTTGCCAGTGTC
	R: GCCTCTCTTCCCACTGTATG
*MMP7*	F: GAATGTTAAACTCCCGCGTC
	R: CGATCCACTGTAATATGCGGTA
*GAPDH*	F: GTGATGCTGGTGCTGA
	R: GCTAAGCAGTTGGTGG
*L. acidophilus*	F: AATTCTCTTCTCGGTCGCTCTA
	R: CCTTTCTAA GGAAGCGAAGGAT

#### Investigating the expression of DEGs in patient tissues by RT-qPCR

We obtained histopathological samples from twelve patients diagnosed with CRC (adenocarcinoma) and their adjacent normal tissues. These samples were collected from the Biobank of Shahid Beheshti University of Medical Sciences. The patients had an average age of 61.41 ± 10.99 years, comprising eight males and four females with severe adenocarcinoma. Total RNA was extracted from the tissue samples by Trizol reagent (Thermo Fisher Scientific, USA), and the RNA’s quantity and quality were assessed using a NanoDrop spectrophotometer (Biotek Instruments, USA) and standard agarose gel electrophoresis. Using the cDNA Synthesis Kit (Thermo Fisher Scientific, USA) and following the manufacturer’s instructions, the RNA was reverse-transcribed into complementary DNA (cDNA). Real-time quantitative PCR (RT-qPCR) was conducted using the RealQ Plus Master Mix Green (Amplicon, Denmark) and the Applied Biosystems 7500 Real-time PCR system (Thermo Fisher Scientific, Inc., USA). The primer sequences employed for RT-qPCR are provided in Table 1. The expression levels were normalized using GAPDH as an endogenous control, and through the 2^−ΔΔCT^ method, the relative expression levels were calculated.

#### Investigating the anti-proliferation and anti-migration effect of *L. acidophilus* postbiotics on HT-29 cell line

The *L. acidophilus* strain (ATCC4356) used in the study was provided from the Persian-type culture collection (Iranian Research Organization for Science and Technology, Tehran, Iran). Respective *L. acidophilus* [1% (v/v)] was grown in de Man, Rogosa, and Sharpe (MRS) broth-rich cysteine (Merck, Germany) for 48 h at 30 °C under anaerobic conditions. Subsequently, 200 µL of 10^8^ CFU/mL *L. acidophilus* bacteria starter culture were inoculated in 500 mL MRS broth-rich cysteine and incubated at 37 °C for 24 h without agitation. Once it reached the late-exponential growth phase at 2.9 × 10^9^ CFU/mL, live *L. acidophilus* was separated from the MRS-conditioned supernatant through a centrifugation step at 10,000×*g* for 15 min at 4 °C, and the supernatant was collected as postbiotics metabolite. Then, the pH of the postbiotics was brought to a physiological range (pH 7.2–7.4) by employing 5 M sodium hydroxide, and the solution was filtered through a 0.22 μm polyethersulfone membrane syringe filter (Millipore, USA). This process was carried out prior to conducting the anti-proliferation and anti-migration assays^28^.

HT-29 cancer cells and human dermal fibroblast (HDF) normal cells were plated onto 96-well microplates (6 × 10^3^ cells per well for HT-29 and 1 × 10^4^ cells per well for HDF) and incubated at 37 °C in a 5% CO_2_ incubator for 3-(4,5-dimethylthiazol-2-yl)-2,5-diphenyl-2H-tetrazolium bromide (MTT) assay. After 24 h, the cells were treated with different concentrations of postbiotics produced by *L. acidophilus* which was applied in a complete growth medium (ranging from 0.62 to 40% v/v) and incubated at 37 °C for 24, 48, and 72 h. After each respective incubation interval, cells were treated with MTT (Sigma-Aldrich, USA) (5 mg/mL) reagent for 4 h. Dimethyl sulfoxide (DMSO, 10%) (Merck, Germany), was added to dissolve the blue formazan crystals. The absorbance of formazan dye was performed at 570 nm, using 630 nm as the reference wavelength. The triplicate samples each time were repeated three times. Finally, the half maximal inhibitory concentration (IC_50_) was calculated by plotting the cell viability percentage against the postbiotic concentration.

The migration potential of *L. acidophilus* postbiotics was assessed through the scratch test. HT-29 and HDF cells were seeded in 12-well plates, with 4 × 10^5^ cells per well for HT-29 and 2 × 10^5^ cells per well for HDF. After 24 h, a straight scratch was created using a 200 μL tip. Cell migration was observed under a microscope at 0–72 h after treatment with 5% v/v of postbiotics. The boundary areas of the scratches were evaluated and captured in photographs. To assess cellular migration, the ratio between the reduced open space after 24–72 h and the initial open space at 0 h was measured using ImageJ software (version 1.52).

#### Cell cycle analysis

HT-29 cells were cultured and treated with *L. acidophilus* postbiotic (at 25% IC_50_ concentration) for 24 h. After the incubation period, the cells were harvested with trypsin, collected in 1.5 mL centrifuge tubes, and washed once with 500 µL of chilled phosphate buffered saline (PBS). Approximately 1 × 10^6^ cells were suspended in 100 μL of PBS and vortexed gently to obtain a mono-dispersed cell suspension with minimal cell aggregation. The suspension was transferred to centrifuge tubes containing 70% ethanol on ice and incubated for 2 h to fix the cells. After fixation, the cells were centrifuged and then suspended in a solution containing PI staining (Sigma-Aldrich, USA), Triton X-100 (Merck, Germany), and DNase-free RNaseA in PBS. The mixture was incubated in the dark at room temperature for 30 min. Finally, the samples were analyzed using flow cytometry^29^.

#### Effects of postbiotics on the gene expression of HT-29 cell line

Total RNA from postbiotics-treated cells (15 × 10^5^ cells per well) was isolated using a Total RNA Extraction kit (Parstous, Iran). The cDNA synthesis was conducted using a cDNA Synthesis Kit (Parstous, Iran). Quantitative RT-qPCR for selected genes was carried out with QuantiTect™ SYBR Green PCR Master Mix (Amplicon, Denmark), and the gene amplification was performed in the ABI Step One™ Real-Time PCR System (Applied Biosystems, CA). We used the *GAPDH* gene to normalize the relative expression for interested genes. The primer sequences used in this study are listed in Table 1. In particular, Relative expression was determined by the 2^(−ΔΔCT)^ technique.

### Statistical analysis

Each experiment was repeated a minimum of three times to ensure reliability. The results were shown as mean ± standard deviation (S.D.). Statistical significance was assessed using Student’s t-test for comparisons between two groups, while ANOVA was employed for comparisons involving more than two groups. A *p*-value less than 0.05 was considered statistically significant.

## Results

### In-silico analysis results

#### Adjacent normal and COAD-derived scRNA-seq profiles display compositional differences

**Figure 2:**
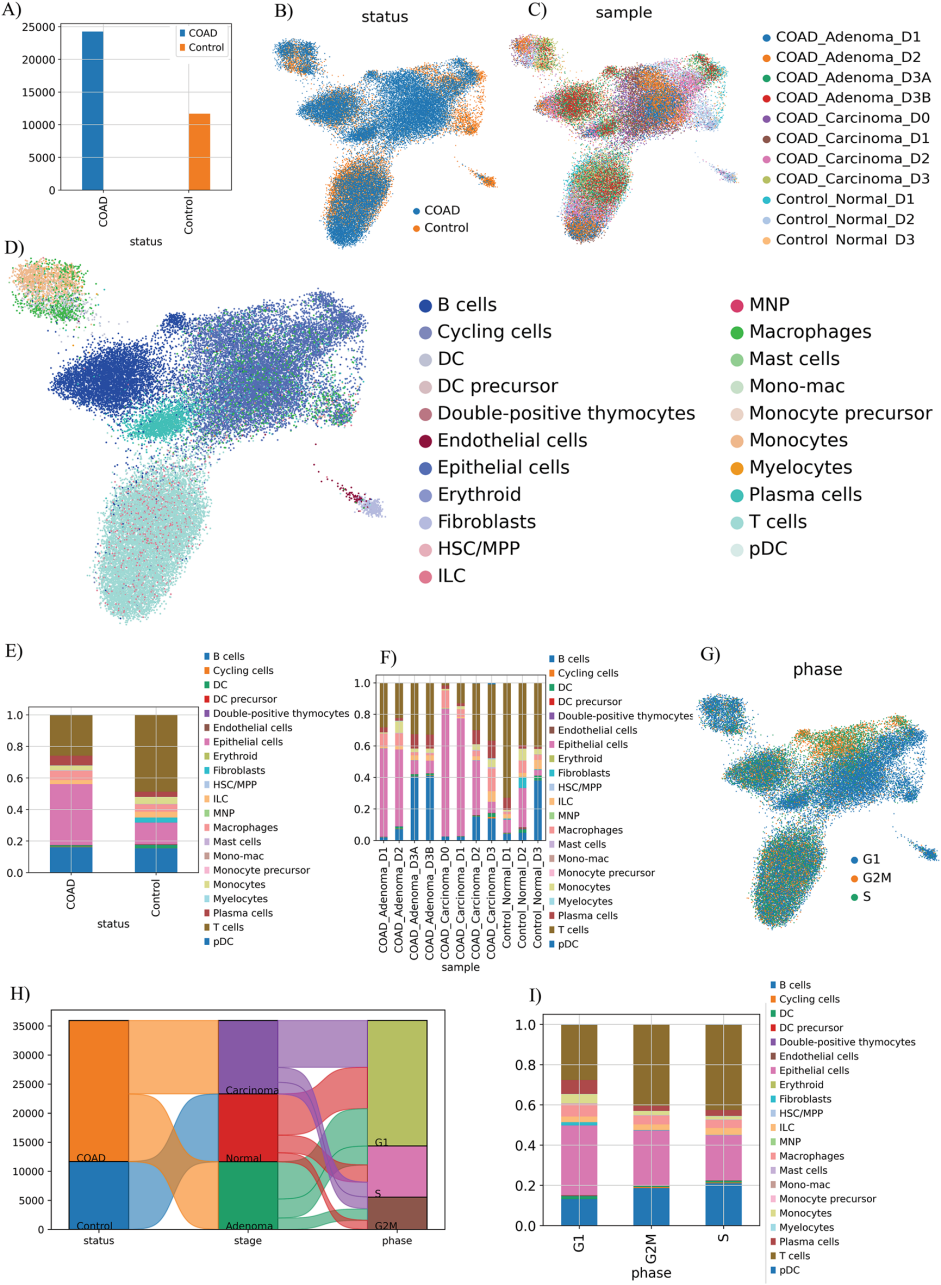
Single-cell RNA sequencing on three normal, four adenoma, and four carcinoma samples. (**A**) Quantification of cells in each statue. (**B**) UMAP visualization of cells between normal and COAD statues (**C**) and between normal, adenoma, and carcinoma samples. Cells are colored according to their statuses and samples, respectively (**D**). UMAP and unsupervised graph-based clustering partitioned cells into 21 clusters according to the expression of canonical gene markers. Cells are colored by cell type. (**E**) The proportion of each cluster between two statuses. (**F**) The proportion of each cluster identified between normal, adenoma, and carcinoma samples. (**G**) The UMAP visualization of cells in different cell phases shows that most cells are in the G1 phase. (**H**) The Sankey plot demonstrates the relative number of COAD and normal cells and their direction in different stages and other cell cycle phases. (**I**) The fraction of each cell type in different cell cycle phases.

A total of 35,951 cells were included in our sc-RNA sequencing analysis after excluding low-quality cells (Fig. S1). These cells were derived from three normal tissues and four samples of adenoma and carcinoma tissues. Cells were classified as originating from normal tissues (*n* = 11,686) and COAD tissues (*n* = 24,265) (Fig. 2A–C). According to the expression of canonical gene markers, the cells were divided into 21 clusters through UMAP and unsupervised graph-based clustering using celltypist ‘Immune_All_High.pkl’ (Fig. 2D). As shown in Fig. 2E,F, there is an abundance heterogeneity in COAD vs normal statues as well as between normal, adenoma and carcinoma samples. The corresponding proportion for each cluster is discrepant. Normal samples comprise a large proportion of T, B, and fibroblast cells, while COAD has a relatively higher proportion of macrophage, epithelial, and fibroblast cells.

Most of the cells are in the G1 phase (Fig. 2G). The Sankey plot demonstrates the relative number of COAD and normal cells and their direction in different stages and other cell cycle phases (Fig. 2H). According to the Sankey plot, most of the cells in each stage of COAD and normal cells were in the G1 phase of the cell cycle. Furthermore, the fraction of each cell type in different cell cycle phases can be visualized in (Fig. 2I).

#### DEGs analysis reveals the presence of the Wnt signaling pathway and four related genes in CRC

**Figure 3:**
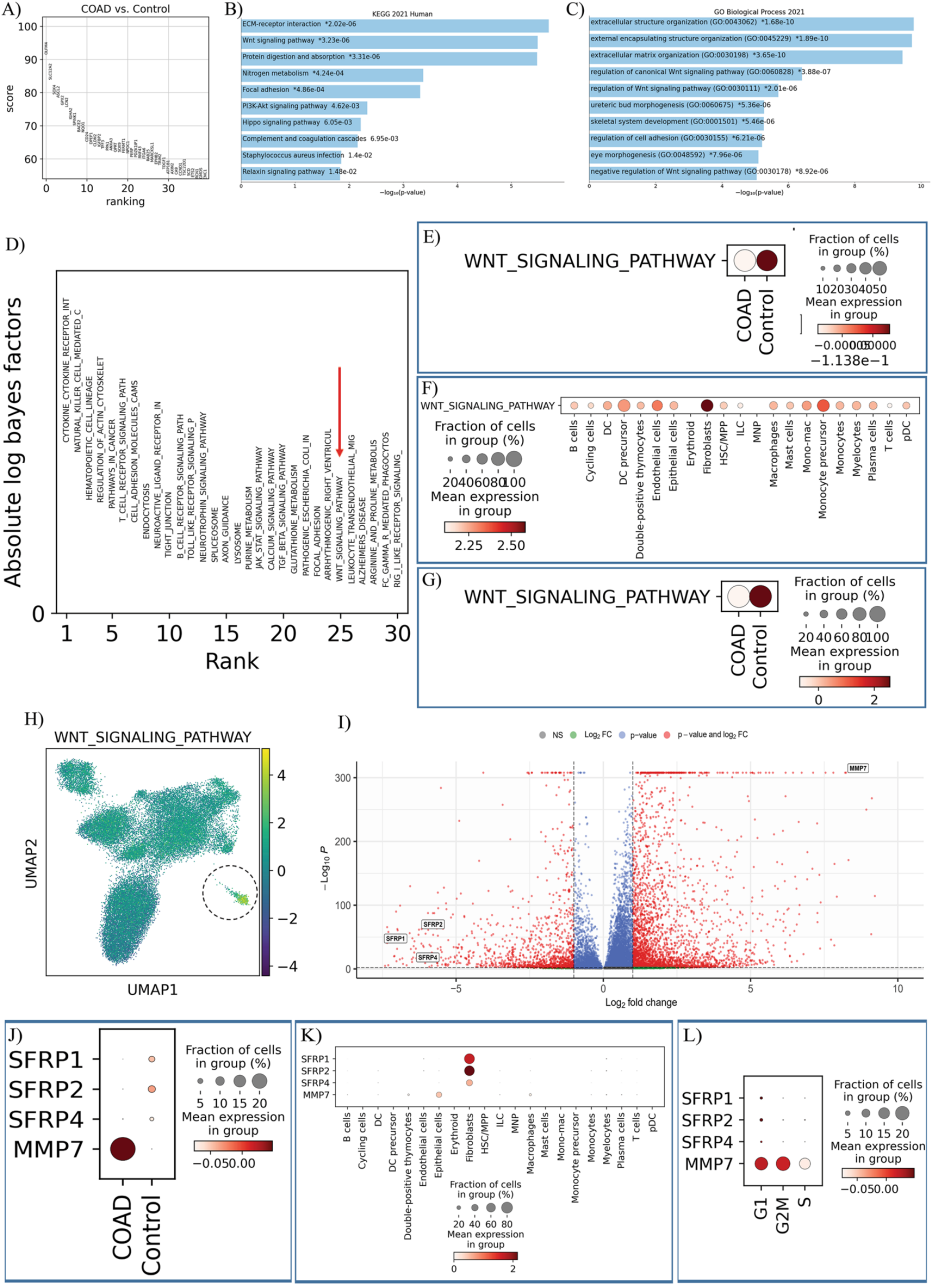
DEG and Enrichment analysis. (**A**) The ranking of identifying total DEGs between the normal and adenocarcinoma samples. (**B,C**) KEGG and GO analysis showed that the Wnt signaling pathway is one of the critical regulators of CRC. (**D**) The ExpiMap also approved the Wnt signaling pathway in COAD. (**E**) The dot plot shows that the Wnt signaling pathway is more activated in control compared to the COAD group in total cells (**F**), which is mainly activated in fibroblast cells. (**G**) The dot plot also shows that the Wnt pathway is mainly activated in fibroblasts of normal cells rather than COAD cells (**H**) which was validated through the UMAP plot. (**I**) The volcano plot shows that four genes, including *SFRP1*, *SFRP2*, *SFRP4*, and *MMP7*, are involved in the Wnt pathway. (**J**) The dot plot visualized the expression patterns of selected DEGs in the COAD tissues compared to the control group. The expression of *MMP7* has increased in the COAD group, while *SFRP1*, *SFRP2*, and *SFRP4* genes had higher levels of expression in the control group (**K**). The dot plot demonstrated that the selected DEGs are mainly expressed in fibroblast. (**L**) The dot plot of the expression patterns of the selected DEGs in different cell cycle phases.

In order to distinguish the total DEGs between the normal and carcinoma samples, we applied the genes that fulfilled the following criteria: adjusted *p*-value < 0.05 and |fold change| ≥ 1. Benjamini–Hochberg was used for adjusting the *p*-value. Based on this criteria, 4562 genes were selected as DEG (1330 downregulated and 3232 upregulated) (Fig. 3A).

Kyoto Encyclopedia of Genes and Genomes (KEGG) and Gene Ontology (GO) analysis was made on the DEGs to find significantly enriched functions with a false discovery rate (FDR) less than 0.05. As a result of Enrichment analyses, the Wnt pathway has appeared as one of the critical regulators of CRC (Fig. 3B,C). Interestingly, ExpiMap also approved the Wnt signaling pathway as one of top30 important pathways in COAD (Fig. 3D). As seen in Fig. 3E, the Wnt signaling pathway has been shown high activity in the control group compared to the COAD in total cells, which it is mainly activated in fibroblast cells (Fig. 3F). Moreover, comparing the Wnt pathway in the fibroblasts of COAD, and normal tissues revealed that this pathway is mainly activated in fibroblasts of normal cells rather than COAD cells (Fig. 3G) which have been validated through our UMAP plot (Fig. 3H).

The results of DEG analysis also showed that four genes, including secreted frizzled-related protein 1 (*SFRP1)*, secreted frizzled-related protein 2 (*SFRP2*), secreted frizzled-related protein 4 (*SFRP4)*, and matrix metallopeptidase 7 (*MMP7*), are involved in the Wnt pathway (Fig. 3I). The dot plot visualized the expression patterns of these DEGs in the COAD tissues compared to the control group. The expression of *MMP7* has increased in the COAD group, while *SFRP1*, *SFRP2*, and *SFRP4* genes had higher levels of expression (Fig. 3J). The dot plot in Fig. 3K also demonstrated that our interesting DEGs, as we expected, are primarily expressed in fibroblast, consistent with our results from the ExpiMap algorithm. Figure 3L demonstrated the expression patterns of the targeted gene in different phases of the cell cycle.

#### The cell–cell interaction of the fibroblasts in adenocarcinoma tissue

In general, fibroblast cells exhibit numerous interactions with various cell types within adenocarcinoma cell populations. Our analysis revealed the highest interaction intensity between fibroblast cells and T cells, as indicated by the Squidpy score. This score was computed by assessing the cumulative ligand and receptor scores across different cell clusters (Fig. S2). Consequently, our research focused on dissecting the molecular and cellular interactions within these two cell types. Among these interactions, several pairs played a pivotal role in the communication between fibroblasts and T cells. These key communication pairs included APP/CD74, B2M/CD3D, B2M/KLRD1, LGALS1/CD69, and TIMP1/CD63 which the interaction between B2M/CD3D was the strongest one (Fig. S2).

#### Characterization of selected DEGs using colon spatial transcriptome data

**Figure 4:**
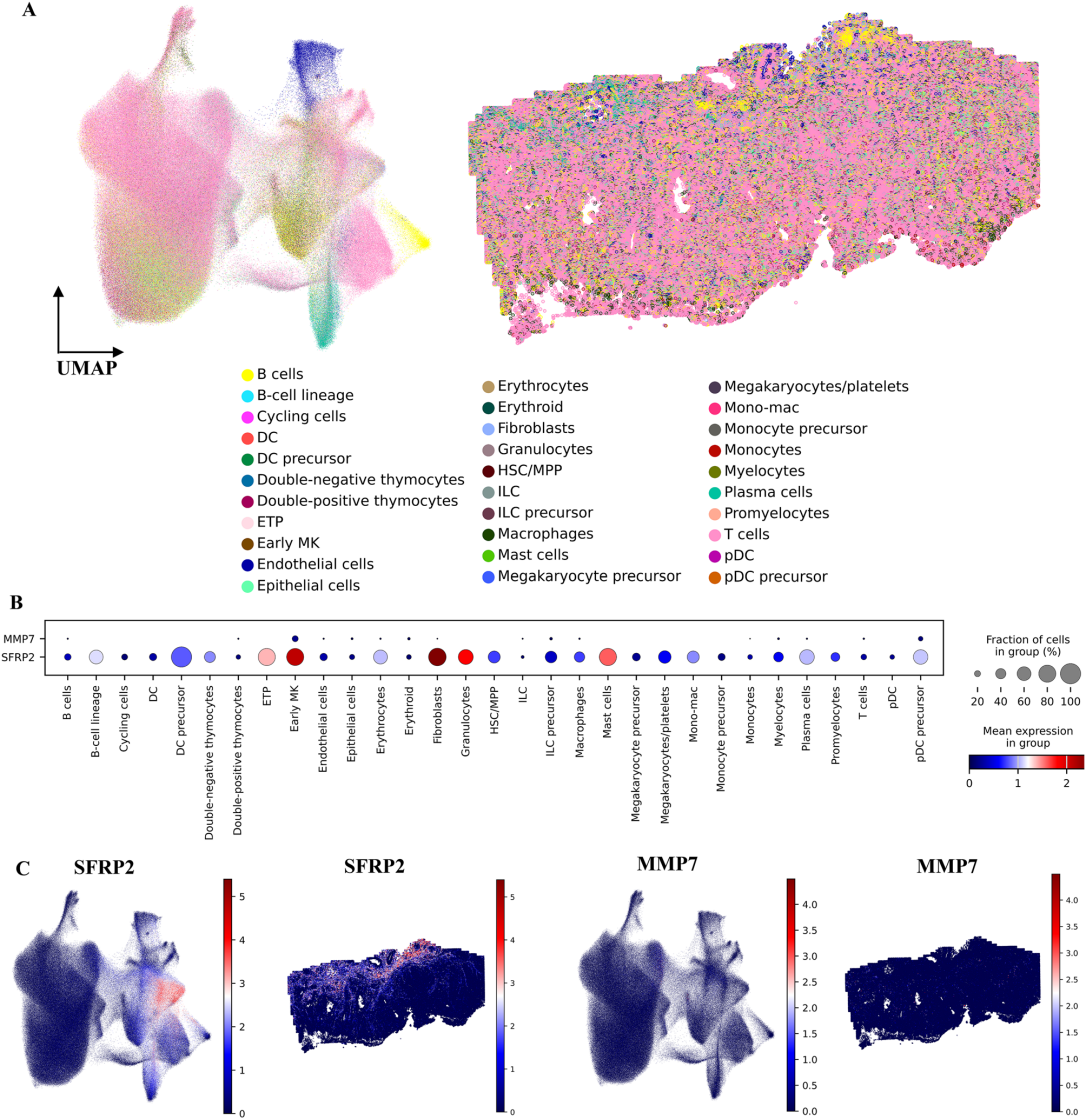
Spatio-temporal analysis of colon cancer Vizgen MERSCOPE. (**A**) UMAP and Spatial distribution of cell types in Colon TME. (**B**) Dotplot visualization of *SFRP2* and *MMP7* in Spatial dataset cell types. (**C**) UMAP and Spatial visualization of *SFRP2* and *MMP7* in Colon TME.

We used the Vizgen MERSCOPE public colon cancer dataset with 817,588 cells to clarify the expression pattern of chosen DEGs in Colon cancer TME. The scanpy normalize total function was used to do data normalization. Squidpy was used to visualize the data^30^. The celltypist Immune high model was then utilized for cell type annotation (Fig. 4A). Each spot of Vizgen MERSCOPE contains 500 genes; based on this, we could only evaluate the expression patterns of *SFRP2* and *MMP7* (Fig. 4B). Following Fig. 3K, spatial data analysis revealed that *SFRP2* is almost expressed in fibroblast cells. *MMP7*, on the other hand, did not reveal a cell type-specific expression pattern (Fig. 4C).

### Characterization of selected DEGs using bulk and cohort datasets

**Figure 5:**
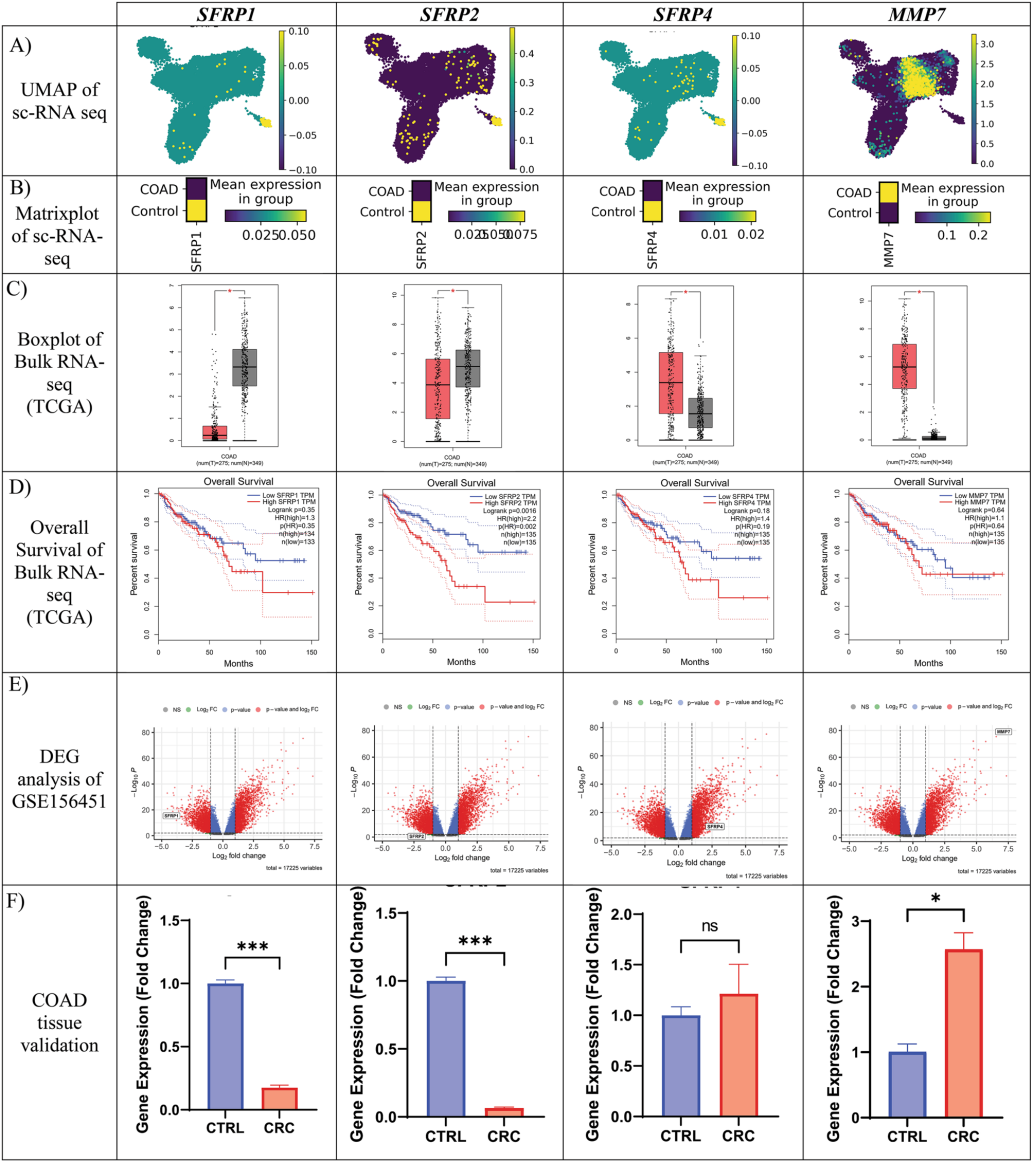
Characterization of selected DEGs across external datasets and tissue validation. (**A**) The UMAP plots and (**B**) matrix plots show the expression of targeted genes in total cells and COAD compared to control groups. (**C**) The box plots and (**D**) overall survival rate of the interested genes from the TCGA-GTEx projects for external validation from the online GEPIA database. (**E**) The volcano plots from bulk analysis represent selected genes. (**F**) Gene expression levels of *SFRP1*, *SFRP2*, *SFRP4*, and *MMP7* in twelve carcinoma vs. normal adjusted tissues. Y-axis represents the fold change in the expression of the target gene in CRC compared with the control group. *Significant difference compared with the control group. (2^(−ΔΔCt)^ = 1 in untreated group). (*p* < 0.05, *p* < 0.01 and, *p* < 0.001 respectively *, **, ***).

The UMAP plots (Fig. 5A) and matrix plots (Fig. 5B) show the expression of targeted genes in total cells and COAD compared to control groups, respectively. The intensity of the yellow color indicates the level of expression for predicted genes. We also used The Cancer Genome Atlas-Genotype Tissue Expression (TCGA-GTEx) projects for external validation via the online GEPIA database. The box plots displayed a decrease in the expression of *SFRP1* and *SFRP2*, along with an increase in *SFRP4* and *MMP7*, in the CRC samples compared to the adjacent normal tissues (Fig. 5C). Additionally, we analyzed the overall survival rate of the interested genes, revealing that *SFRP1*, *SFRP2*, *SFRP4*, and *MMP7* could play crucial roles in the overall survival rate of CRC patients. However, only p(hazard ratio) for *SFRP2* was significant (*p*-value = 0.002) (Fig. 5D). Furthermore, we conducted bulk analysis on the GSE156452 dataset from GEO to visually represent our selected genes using volcano plots, where *SFRP1* and *SFRP2* were found to be downregulated, while *SFRP4* and *MMP7* were upregulated (Fig. 5E). This result was inconsistent with TCGA dataset.

### In vitro analysis results

#### Absolute RT-qPCR of total *L. acidophilus* load

The mean quantity of *L. acidophilus* copy numbers was calculated for the two examined groups (*p*-value < 0.001). A significant statistical distinction was observed when comparing the healthy group to CRC patients (Table 2).

**Table 2:** Copy number of *L. acidophilus* bacteria in stool samples taken from CRC patients and healthy individuals.

Microorganism	Type of sample	Minimum	Maximum	Mean
*L. acidophilus*	Healthy	2.439747 × 10^7^	2.255690 × 10^8^	9.981064 × 10^7^
	CRC	2.885416 × 10^5^	8.683294 × 10^6^	5.385177 × 10^6^

#### Evaluating the expression of Wnt signaling pathway genes in patient tissues by RT-qPCR

The expression level of the *SFRP1*, *SFRP2*, *SFRP4*, and *MMP7* was evaluated using RT-qPCR. Inconsistent with in silico results, the expression of *SFRP1* (5.7-fold down, *p*-value < 0.001) and *SFRP2* genes (15.3-fold down, *p*-value < 0.001) in CRC samples exhibited a substantial decrease compared to the adjacent normal tissues. However, the expression of *MMP7* (2.57-fold up, *p*-value < 0.05) and *SFRP4* (1.2-fold up, *p*-value = 0.42) had upregulated in the CRC samples. No significant changes were observed in *SFRP4* expression among CRC and control samples (Fig. 5F).

#### *L. acidophilus* postbiotics had anti-proliferation and anti-migration effects on HT-29 cell line

**Figure 6:**
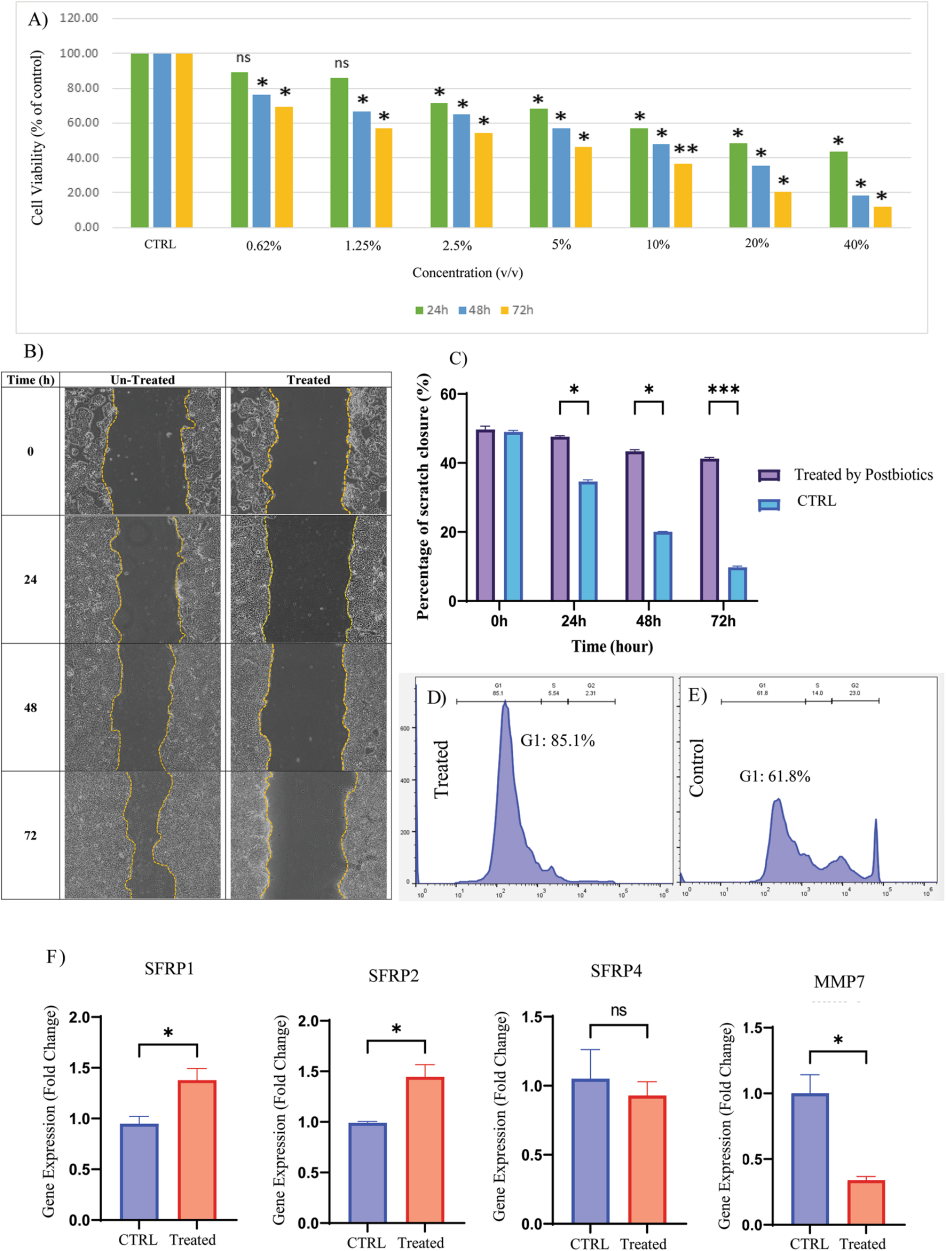
Experimental validation of selected genes. (**A**) MTT assay showing the effect of *L. acidophilus* on reducing the viability of HT-29 cell line. MTT analysis indicates that postbiotics had significant cytotoxic effects (IC_50_ value of 20% v/v) on HT-29 cells compared to the untreated group. (**B**) Scratch test showing the effects of *L. acidophilus* postbiotics on reducing the migration of HT-29 cell line. Under 80% confluence, a scratch was made on wells using a sterile yellow-colored pipette tip. Subsequently, 25% IC_50_ concentrations of postbiotics were added to the cells. Pictures were taken at 0 and 72 h, and the amount of migration at 0 and 72 h was calculated. (**D,E**) Flow cytometry analysis of cells treated by postbiotic showed a substantial increase in the proportion of treated cells in the G1 phase compared to the control group. (**F**) Gene expression levels of *SFRP1*, *SFRP2*, *SFRP4*, and *MMP7* in HT-29 cell line in response to *L. acidophilus* postbiotics treatment. Y-axis represents the fold change in the expression of the target gene compared with the untreated group. *Significant difference compared with the untreated cells (2^(−ΔΔCt)^ = 1 in untreated group). (*p* < 0.001, *p* < 0.01 and *p* < 0.05 respectively ***, **, *).

To examine the anti-proliferation and anti-migration effects of *L. acidophilus* postbiotics on the HT-29 cell line, we cultured the cells and treated them with different concentrations of postbiotics for different times (0.62–40% v/v; 0–72 h). The effect of postbiotics on cancer cell proliferation was assessed by MTT assay. Postbiotic treatments lead to significantly decreased cell proliferation after 24, 48, and 72 h in a dose- and time-dependent manner compared to the control group without postbiotics. The IC_50_ value for the HT-29 cells treated with postbiotics for 24 h was 20% v/v. As shown in (Fig. 6A), the IC_50_ values of postbiotics significantly decreased after different times (24–72 h). Although postbiotics exhibited significant cytotoxic effects on HT-29 cells, no IC_50_ value was detected for HDF cells, except for limited cytotoxicity of 40% (v/v) when treated with *L. acidophilus* for 72 h (results not shown). The scratch test was used to measure the cell migration in the HT-29 cell culture plate with/without treatment with postbiotics (Fig. 6B). Data indicated that at 5% v/v dose of postbiotics, the *L. acidophilus* extraction considerably reduces the migration of HT-29 cells after 0–72 h compared to the untreated control group (Fig. 6C).

#### *L. acidophilus* postbiotic induces G1 cell cycle arrest

Our flow cytometry analysis revealed that postbiotic treatment had a significant impact on the cell cycle dynamics. Notably, postbiotics induced a pronounced inhibition of the G1 phase, leading to a substantial increase in the proportion of treated cells in the G1 phase (Fig. 6D) compared to the control group (Fig. 6E). Concurrently, we observed a marked reduction in the proportions of cells in the S and G2M phases, suggesting that postbiotic treatment primarily affects the progression of cells through the G1 phase of the cell cycle.

#### *L. acidophilus* postbiotics dysregulate the Wnt signaling pathway in HT-29 cell line

RT-qPCR data demonstrated that treating the HT-29 cells with postbiotics effectively decreased the expression levels of *MMP7* (2.9-fold down, *p*-value < 0.01) while increasing the expression of *SFRP1* (1.5-fold-up, *p*-value < 0.05), and *SFRP2* (1.4-fold up, *p*-value < 0.01) compared to untreated cells. Accordingly, no significant changes were noticed in *SFRP4* expression among both controls and treated cells (1.1-fold down, *p*-value = 0.54) (Fig. 6F).

## Discussion

In this investigation, we comprehensively studied the expression of important genes in CRC using state-of-the-art scRNA-seq data analysis. To accomplish this, we acquired the GSE8161277 scRNA-seq dataset from the GEO database and performed analysis employing the Scanpy pipeline. Our investigation encompassed four adenoma samples, four carcinoma samples, and three samples of normal adjacent tissue, ensuring a robust representation of the CRC microenvironment. To gain deeper insights into the functional implications of DEGs, we carried out GO and KEGG enrichment analyses. Remarkably, our findings underscore the significant involvement of the Wnt signaling pathway in CRC progression.

Additionally, the cell–cell interaction analysis showed that fibroblasts and T cells exhibit a notably strong interaction, primarily facilitated by the Beta-2 Microglobulin (B2M)/CD3D axis. B2M is an important subunit of class I major histocompatibility complex (MHC), which plays a substantial biological role in tumorigenesis and immunological activity^31^. CD3 is a part of the CD3 complex, a crucial component of the T-cell receptor (TCR) complex found on the surface of T cells^32^. The strong B2M/CD3D interaction between fibroblasts and T cells suggests a complex interplay within the TME. Their interaction in adenocarcinoma may indicate their involvement in modulating immune responses in the tumor context. This interaction could potentially influence T cell activation, function, or recruitment within the tumor, impacting the immune response against cancer cells^33^.

Afterward, we selected four notable genes, *SFRP1*, *SFRP2*, *SFRP4*, and *MMP7*, from within the Wnt signaling pathway for further characterization. To reinforce the validity of our findings, we analyzed a bulk RNA-seq dataset and integrated TCGA data to verify the expression patterns of the selected DEGs. Encouragingly, the expression profiles obtained from the scRNA-seq and bulk analysis demonstrated a remarkable concurrence, validating the robustness of our findings. However, it is noteworthy that the gene *SFRP4* exhibited an unexpected upregulation in COAD samples in the bulk analysis, deviating from our initial expectations.

To further strengthen our observations, we conducted RT-qPCR experiments on tissue samples obtained from twelve Iranian adenocarcinoma patients alongside their respective normal adjacent tissues. Also, these genes were treated with *L. acidophilus* postbiotics in the HT-29 cell line, shedding light on their intricate roles in the disease context.

These experimental results aligned closely with our bulk in-silico findings, reinforcing our comprehensive analysis’s reliability. It is worth mentioning, however, that the *SFRP4* gene displayed no significant expression alterations in our RT-qPCR analysis, deviating from the anticipated trends.

The Wnt signaling pathway is a complex protein interaction system with a central role in various biological processes. While it is primarily associated with embryonic development and cancer, it regulates normal physiological processes in adults. Specifically, during the development process, the cell pluripotency and the direction of cell differentiation are regulated by the canonical Wnt signaling pathway^34,35^. The Wnt signaling pathway encompasses various components whose dysregulation in their expression is believed to contribute to several disease conditions, including CRC. These components have been recognized as reliable biomarkers and promising targets for cancer treatment^36^.

SFRPs are a group of glycoproteins that their primary function is to antagonize the activity of Wnt signaling^37^. SFRPs possess a domain rich in cysteine, resembling the domain responsible for binding Wnt ligands in the frizzled (Fz) membrane receptor. Consequently, they compete with Fz receptors to bind to Wnt ligands or directly interact with Wnts. This interaction leads to the stabilization and increased levels of the transcription factor β-catenin, which plays a central role in regulating the transcription of genes targeted by the Wnt pathway^34^. Therefore, any abnormal alterations in SFRPs can lead to the activation of the Wnt pathway irregularly, deviating from the normal signaling pattern^38^.

The *SFRP1* and *SFRP2* constrain the Wnt pathway by binding to and sequestering Wnt ligands, preventing them from activating the Fz receptors on the cell surface. This, in turn, leads to the inhibition of downstream signaling events, including the localization of β-catenin in the nucleus and activation of Wnt target genes such as *MMP7* and cMYC^39^. Many studies have shown that the expression of *SFRP1* and *SFRP2* is often downregulated in CRC, leading to dysregulation of the Wnt signaling pathway. This downregulation may occur through various mechanisms, including genetic mutations, epigenetic changes, and altered protein expression^40–43^. Unlike *SFRP1* and *SFRP2*, which are negative regulators of the WNT pathway, *SFRP4* has been found to be overexpressed in CRC, suggesting potential distinct biological roles for it^44^. Some studies have indicated that *SFRP4* may not be a significant inhibitor in antagonizing the Wnt pathway because of the differences in DNA hypermethylation and expression compared to other members of the SFRP family^45^. However, an alternative study using a large-scale tissue microarray showed independent co-expression of *SFRP4* and *p53*, and the absence of *SFRP4* expression was associated with the negative expression of *COX-2* and *MLH1*. Therefore, the overexpression of *SFRP4* in CRC remains unclear, so that *SFRP4* might have a role at the intersection of the Wnt pathway and other signaling pathways^46^.

*MMP7*, also known as Matrix metalloproteinase-7, is an enzyme that degrades extracellular matrix (ECM) proteins. It is highly expressed by epithelial tumor cells in invasive CRC compared to normal cells and is associated with distant metastasis. *MMP7* is a downstream target gene of the Wnt signaling pathway, which is frequently aberrantly activated in CRC^47^. This results in beta-catenin’s stabilization and nuclear translocation, followed by the initiation of target gene transcription such as *MMP7*^48^. The upregulation of *MMP7* in CRC promotes ECM degradation, invasion, and metastasis of CRC cells and activates other signaling pathways. As a result, targeting *MMP7* could be a potential therapeutic strategy for CRC^49^.

Numerous studies have highlighted the intricate connection between microbiota and human health^6^. Notably, bioactive substances like postbiotics generated by probiotic bacteria have shown the potential to influence the efficacy of cancer treatment and mitigate the adverse effects of traditional therapies in cancer patients. These properties are attributed to their anti-proliferative, anti-inflammatory, and anti-cancer properties^18,50^. Various reports have indicated that postbiotics, with their distinct characteristics, have gained significant scientific interest and are considered a novel adjunctive therapy approach in cancer patients^6,51^.

In this regard our research has revealed a connection between *L. acidophilus* and CRC progression, while also pointing to promising therapeutic options. The results revealed that postbiotics derived from *L. acidophilus* exhibit the ability to inhibit the proliferation of CRC cells by influencing the Wnt signaling pathway and exerting an anti-migration effect on them. Our results were further complemented by flow cytometry data, demonstrating the ability of *L. acidophilus* postbiotics to arrest CRC cells in the G1 phase of the cell cycle. These findings underscore the importance of balanced gut microbiota, including sufficient *L. acidophilus* levels and the associated postbiotic production, in the fight against CRC—a notion reinforced by our validation through absolute RT-qPCR results. Quantifying the low *L. acidophilus* levels in CRC patients compared to healthy individuals may lead to a consequential reduction in postbiotics production. This decrease in postbiotic production can have significant implications for CRC development through dysregulation of many pathways, such as the Wnt pathway. Our results showed the relationship between *L. acidophilus*, CRC progression, and promising therapeutic strategies.

In line with our results, several studies confirmed the potential role of postbiotics in preventing and treating cancer, particularly CRC. By this means, Salami et al. demonstrated that *Lactobacillus rhamnosus GG* could selectively reduce the viability of cancer cells, such as HT-29 cells, in a dose-dependent way, with a mitotic arrest the cell cycle in the G2/M phase^28^. Some studies, such as Shyu et al., focus on the anti-proliferative and apoptosis effects of Lactobacillus species in various cell lines. They showed that the postbiotics extracted from Lactobacillus species driven from dairy products have anti-cancer effects on various cancer cell types, including CRC cells (HCT116 and HT-29), through the increased expression of early apoptotic-promoting genes (*cfos*, *cjun*) and decreased the expression of pro-inflammatory cytokine genes (*IL-β*, *TNF-α*)^52^. The anti-cancer effect of postbiotics by induction of apoptosis was also confirmed by Lee et al. They showed that the *Lactobacillus fermentum* postbiotics exerts its anti-cancer effects by induction of apoptosis in three-dimensional (3D) spheroids of CRC cells in vitro. In addition, the expression of apoptosis genes was significantly changed after treatment with postbiotics in a 3D system^53^. At the same time, some in vivo studies have shown that probiotics in animal models have inhibitory activity against CRC. In this way, Chen et al. found that *Clostridium butyricum,* as a probiotic bacteria, could prevent the development of intestinal tumors in mice models by modifying the Wnt pathway and gut microbiota. Therefore, they suggested the possible efficacy of butyrate-producing bacteria against adenocarcinoma^54^.

Regarding the molecular mechanism of butyrate as a SCFAs in CRC progress, it has been demonstrated that butyrate acid has the ability to suppress the expression of Neuropilin-1 (NRP-1) by inhibiting the activity of specificity protein 1 (Sp1). This mechanism ultimately leads to inhibiting angiogenesis and metastasis in CRC cells^55,56^. Furthermore, SCFAs can hinder the proliferation of CRC cells by activating the extracellular signal-regulated kinases (ERK)/Mitogen-activated protein kinase (MAPK) signaling pathway, thereby stimulating the expression of endocan^56,57^. Conversely, SCFAs induce apoptosis in CRC cells by increasing the expression of Bcl-2-associated X protein (*Bax*)^58,59^. The use of postbiotics in treating CRC is still in its early stages, so further research is required to completely understand the molecular mechanisms underlying their effects and develop targeted therapies to improve patient outcomes. Although our study indicates the anti-cancer and anti-migration effects *of L. acidophilus* postbiotics on HT-29 cells, other types of CRC cells may not behave similarly. Therefore, more in-vitro and in-vivo study is needed to evaluate postbiotics’ anti-tumor, safety, and side effects. These efforts will shed light on the future of cancer therapies.

While this investigation provides valuable insights into the potential impact of *L. acidophilus* postbiotics, it is essential to acknowledge certain limitations that should be considered when interpreting the results. Our in vitro experiments with *L. acidophilus* postbiotics demonstrated potential anti-cancer effects on the HT-29 cell line, it is important to recognize that the behavior of CRC cells can vary widely, and the effects observed in this study may not necessarily extend to all CRC cell types. Additionally, the translation of these findings to in vivo models and ultimately to clinical applications would require further investigation, including studies assessing the safety and potential side effects of postbiotic interventions.

## Conclusion

Our study used scRNA-seq analysis to uncover the significant role of four specific genes (*SFRP1*, *SFRP2*, *SFRP4*, and *MMP7*) in the Wnt signaling pathway in CRC compared to normal tissue. Our RT-qPCR results on tissue samples confirmed the in silico findings, showing decreased expression of *SFRP1* and *SFRP2* genes, increased expression of *MMP7*, and relatively unchanged expression of *SFRP4*. Additionally, we confirmed the anti-proliferation and anti-migration effects of *L. acidophilus* postbiotics on HT-29 cell lines in a dose and time-dependent manner. We concluded that *L. acidophilus* postbiotics may exert its anti-tumor effects by influencing critical genes in the Wnt signaling pathway, such as *SFRP1*, *SFRP2*, and *MMP7*. However, there were no significant changes in *SFRP4* gene expression. These findings contribute valuable knowledge to exploring postbiotics from probiotic bacteria as potential agents against cancer growth and migration.

### Supplementary Information

Supplementary Figures.

## Data Availability

The datasets analyzed during the current study are available in the Gene Expression Omnibus (https://www.ncbi.nlm.nih.gov/geo/query/acc.cgi?acc=GSE161277) and (https://www.ncbi.nlm.nih.gov/geo/geo2r/?acc=GSE156451) repository.
