# Author Correction: Characterization of Wnt signaling pathway under treatment of *Lactobacillus acidophilus* postbiotic in colorectal cancer using an integrated in silico and in vitro analysis

**DOI:** 10.1038/s41598-024-57127-6

**Published:** 2024-03-15

**Authors:** Nafiseh Erfanian, Saeed Nasseri, Adib Miraki Feriz, Hossein Safarpour, Mohammad Hassan Namaei

**Affiliations:** 1 Student Research Committee, Birjand University of Medical Sciences, Birjand, Iran; 2 Cellular and Molecular Research Center, Birjand University of Medical Sciences, Birjand, Iran; 3 Infectious Diseases Research Center, Birjand University of Medical Sciences, Birjand, Iran

Correction to: *Scientific Reports* 10.1038/s41598-023-50047-x, published online 27 December 2023

The original version of this Article contained an error in the spelling of the author Mohammad Hasan Namaei which was incorrectly given as Mohammad Hassan Namaie.

The original Article has been corrected.

