# Supplementary material for: Characterization of Wnt signaling pathway under treatment of Lactobacillus acidophilus postbiotic in colorectal cancer using an integrated in silico and in vitro analysis
Source: Sci Rep. 2023 Dec 27;13:22988. doi: 10.1038/s41598-023-50047-x (PMC10752892; doi:10.1038/s41598-023-50047-x)
Supplement: Supplementary file 1 — Supplementary Figures. [file 41598_2023_50047_MOESM1_ESM.docx]

**Supplementary files:**


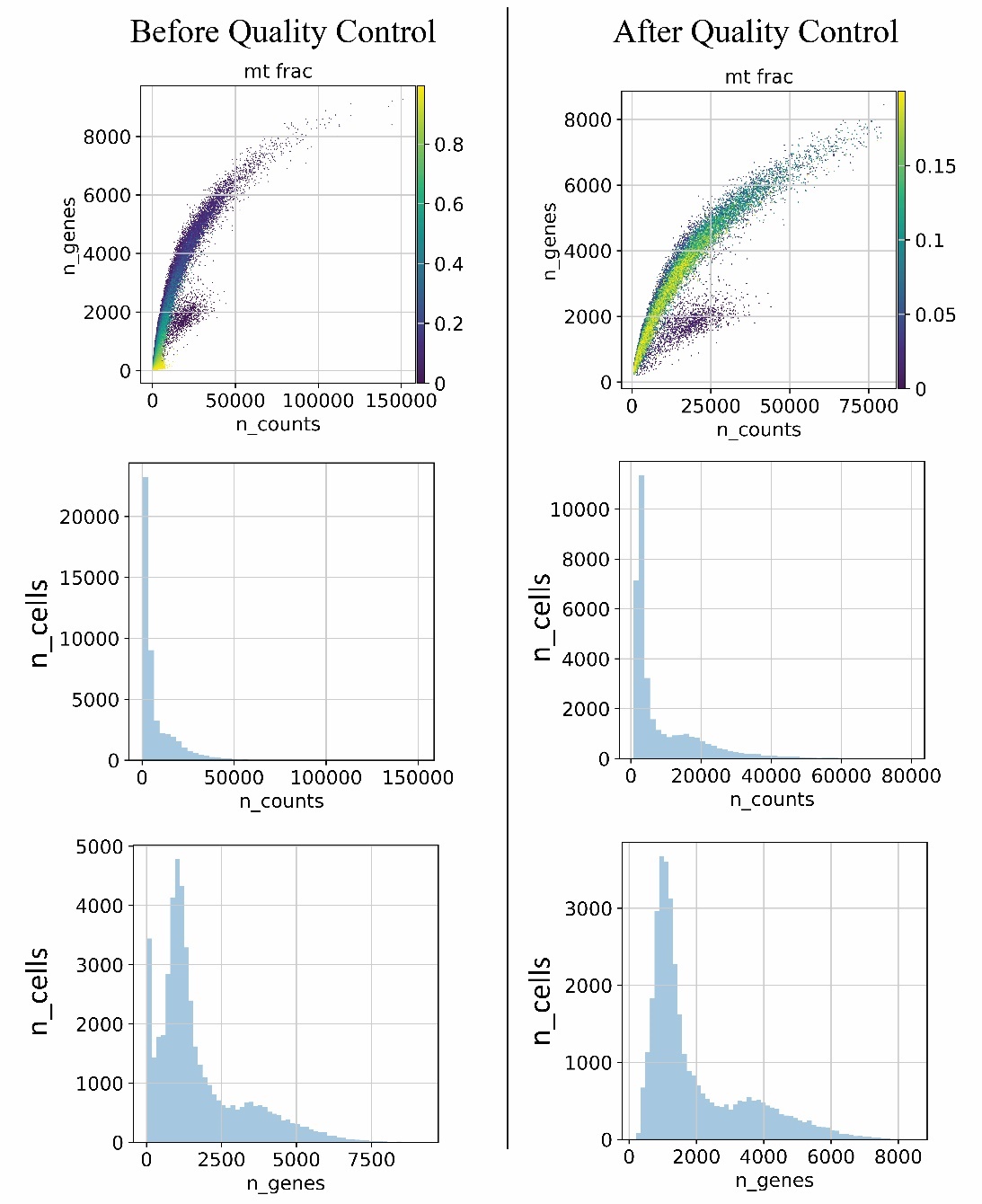


**Figure S1:** Quality control of cells and genes.


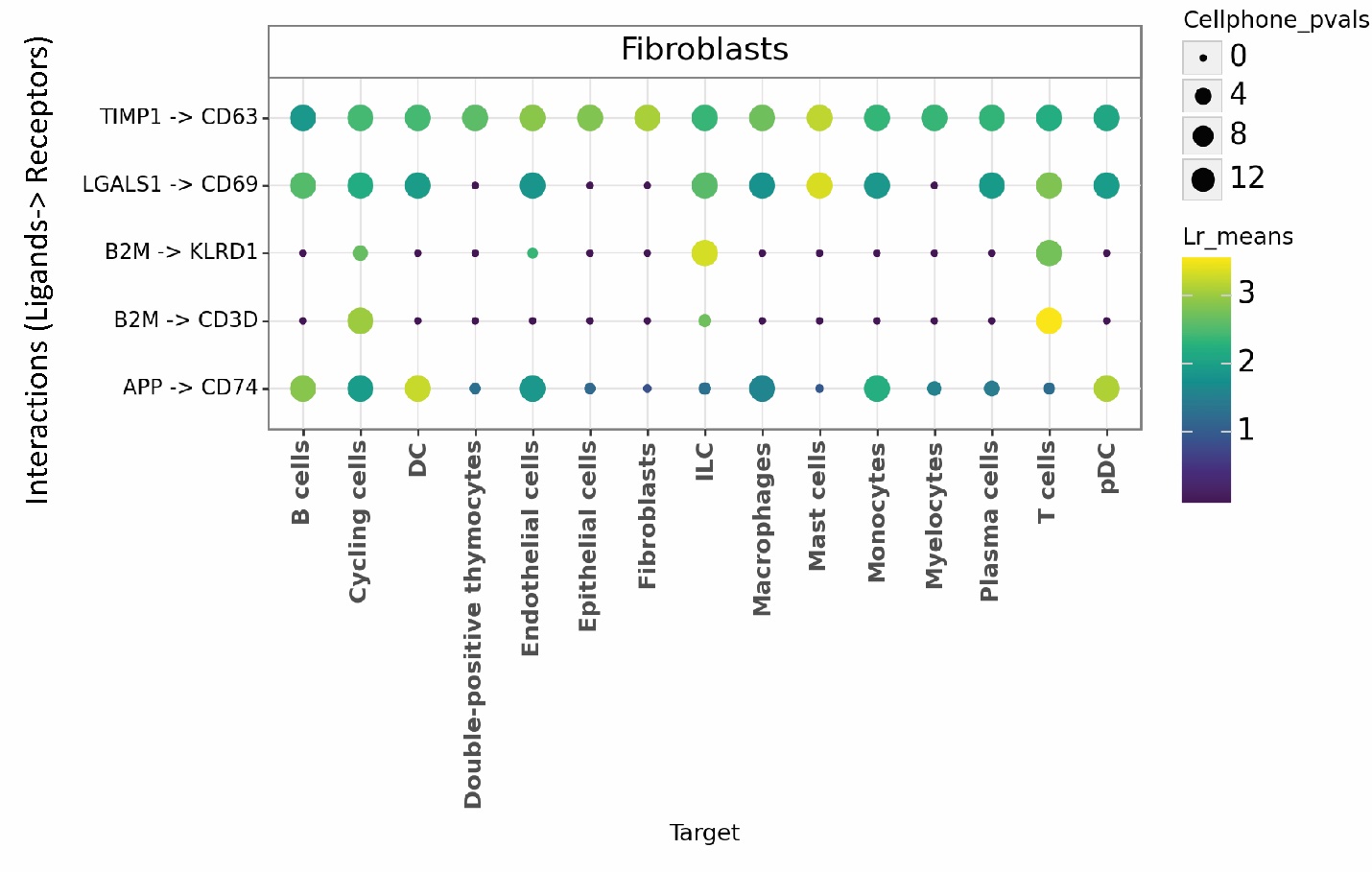
**Figure S2:** The cell-cell interaction of the fibroblasts in adenocarcinoma tissue
